# An Orthogonal Synthetic Approach to Nonsymmetrical Bisazolyl 2,4,6-Trisubstituted Pyridines

**DOI:** 10.3390/molecules27051746

**Published:** 2022-03-07

**Authors:** Arturo Gamonal Ruiz-Crespo, Laura Galán-Fernández, Paloma Martínez-Martín, Juan Carlos Rodríguez-Ubis

**Affiliations:** Departamento de Química Orgánica, Facultad de Ciencias, Universidad Autónoma de Madrid, 28049 Madrid, Spain; arturo.gamonalrc@gmail.com (A.G.R.-C.); laura.galan@inv.uam.es (L.G.-F.); paloma.martinez1990@gmail.com (P.M.-M.)

**Keywords:** pyrazolylpyridines, indazolylpyridines, nucleophilic substitution, C–C coupling

## Abstract

A three-step synthetic route giving access to nonsymmetrical bisazolyl 2,4,6-trisubstituted pyridines with different substituents on the pyrazole, indazole, and pyridine heterocycles is described. From the readily available 4-bromo-2,6-difluoropyridine, both fluorine atoms allow for easy selective stepwise substitution, and the bromine atom provides easy access to additional functionalities through both Suzuki and Sonogashira Pd(0) cross-coupling reactions. These synthons represent optimal structures as building blocks in complexation and metalloorganic structures for the tuning of their chelating and photophysical properties.

## 1. Introduction

Poly-*N*-heterocyclic frameworks with extended electronic delocalization have been traditionally used as effective and stable ligands for the complexation of transition metal ions. These metal ion complexes, in particular polypyridine ligands such as 2,2′:6,2′′-terpyridine (**tpy**) [1,2,3], have been used in the design of luminescent devices or as sensitizers for light-to-electricity conversion due to their rich photophysical, photochemical [4,5,6], and electrochemical properties [7].

The use of 2,2′:6′,2′′-terpyridines in a wide range of applications and in a variety of research areas has created a sizeable ‘‘pool’’ of different functionalized terpyridines. Therefore, a highly efficient and simple synthesis is as essential as well-defined derivatization at every ring position. While the number of publications concerning applications with terpyridine complexes has increased enormously, comparably the number of reported functionalized 2,2′:6′,2′′-terpyridine derivatives is much lower [1,8], as many of the synthetic methods involve ring assembly reactions, which shorten and hinder access to these interesting tridentate pincer ligands.

Other terdentate pyridine-centered heteroaromatic ligands represent particularly privileged coordination frameworks. Among them, combination with side-by-side azole rings such as pyrazole, indazole, oxazole, triazole, and tetrazole, and flanking to pyridine rings is of great interest [9,10]. The compound 2,6-bis(pyrazol-1-yl)pyridine (**bppy**) has been used as a versatile analogue to the 2,2′:6′,2′′-terpyridine. 

Based on a redox study by Jameson and colleagues on Ru(L_2_)^2+^ complexes, bppy is a weaker **π**-acceptor and **σ**-donor. This is due to the lesser basicity of pyrazole and the higher π* energy of the aromatic system, making **bppy** less effective in stabilizing Ru(II) because of less ligand binding strength [11,12]. However, the synthetic ease with which **bppy** may be derivatized on the 4-position of pyridine [13,14,15], as well as on the 3-, 4-, and 5-positions of the pyrazole rings [16,17], has led to the increasing popularity of **bppy** as a complementary platform for research into new d- and f-block metal complexes [18].

Along with **bppy**, other promising ligands in this class include 2,6-(indazol-1-yl)pyridine (**bipy**), where pyridine has a central role as a coordinating ring for this terdentate supramolecular system. In this context, the synthesis of these terdentate compounds is rather straightforward for the unsubstituted ligands [11,12,19,20] or for the symmetrically substituted pyrazole or indazole rings [16,21], but not so straightforward for ligands with different substituents on the heterocyclic rings. It is even more difficult in regards to access to nonsymmetrical structures with different rings flanking the pyridine central ring or different substituents on each side [17,22,23].

These differently substituted polyheterocycles open the possibility of tuning the physico-chemical properties of their complexes. Their synthesis is very challenging with few examples described in the literature and little access to this type of nonsymmetrical bisazolylpyridine.

These **bppy** and **bipy** scaffolds are particularly attractive in comparison with terpyridine because they are easily altered by varying the substitution pattern based on the different reactivities of the azole rings compared with that of pyridine, opening the possibility of orthogonal synthesis. 

In this communication, we describe a simple and straightforward method for synthetic access to versatile polysubstituted terdentate pyridine-centred heteroaromatic ligands. 

By means of a combination of an azolate nucleophilic substitution reaction and palladium cross-coupling reaction (Suzuki–Miyaura- and Shonogashira-type reactions), different substitutions on the pyridine, pyrazole, and/or indazole rings are possible over the easily available 4-bromo-2,6-difluoropyridine in very mild reaction conditions. 

## 2. Results and Discussion

Based on the pioneering work of Schlosser regarding the reactivity of polyfluoropyridines [24,25,26], the 4-bromo-2,6-difluoropyridine molecule has been described as a good alternative for accessing 2,6-dipyrazole and 2,6-diindazolepyridine derivatives [13,14,27]. The reaction conditions for the nucleophilic substitution of fluorine atoms by pyrazolate or indazolate salts are milder and much more convenient (yield, temperature, and work-up) than those of Jameson’s work on the substitution of 2,6-dibromopyridine towards **bppy [11,12]**. Until recently, our work with biazolylpyridines has used a methodology based on pyrazolate susbstitution on 4-substituted 2,6-dibromopyridine [28,29]; however, the type of substituent at the 4-position has been limited to –OMe, –CN, and amide groups [30], and in other cases, to aromatic substituents, such as phenyl or thiophene, by more tedious synthetic access [31,32].

In the case of 4-bromo-2,6-difluoropyridine **1** being used as the starting material, the unreacted 4-bromine position has been exploited as an excellent functionalization board for the introduction of almost any functional group by palladium-mediated cross-coupling reactions [14].

The selective reactivity of fluorine atoms related to bromine has opened the possibility of a first orthogonal approximation to nonsymmetrical 4-substituted 2,6-bipyrazole (**bppy**), 2,6-biindazole (**bipy**), and 2-indazole-6-pyrazolepyridine (**ippy**) frameworks, which is applied in the preparation of lanthanide luminescent complexes. We are interested in these kinds of complexes for the development of a switchable lanthanide-based bioassay for DNA recognition [33].

The different substituents located on the nonsymmetrical **bppy**, **bipy**, or **ippy** units are employed with different purposes on each azole ring: as an anchoring isothiocyanate group to conjugate the DNA sequence; as an additional lanthanide coordinating group such as a carboxylic acid; and by means of the different electron-demanding groups introduced over the central pyridine ring, the opportunity to play with the photophysical properties of these ligands.

**Scheme 1:**
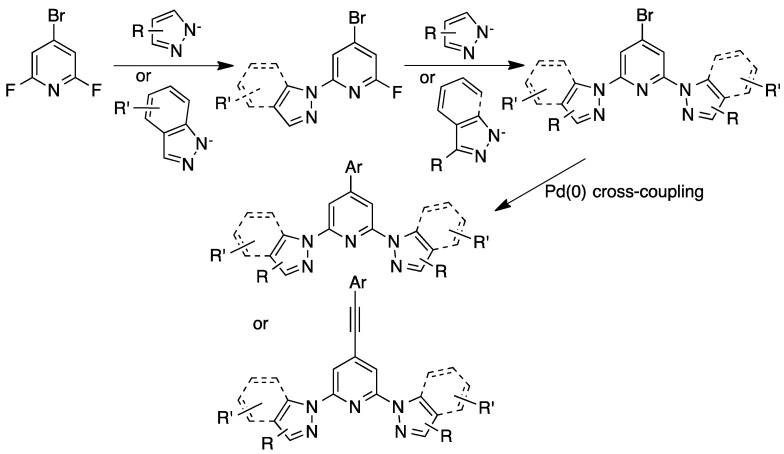
Synthetic access to nonsymmetrical **bppy**, **bipy**, or **ippy** units.

By choosing the appropriate reaction conditions, it is possible to selectively control the introduction of a substituted pyrazole or indazole ring, obtaining mono 2-pyrazole- or 2-indazole-4-bromo-6-fluoropyridine derivatives in good yields. From these compounds, it is possible to perform a second functionalization of the remaining fluorine atom by nucleophilic substitution with an adequately substituted pyrazole or indazole ring. Over the nonsymmetrical 2,6-disubstituted 4-bromopyridine, a cross-coupling reaction over the brominated position gave the terdentate ligand with very high versatile substitution (Scheme 1).

### 2.1. Indazole Pyrazole Pyridine (***ippy***)

**Scheme 2:**
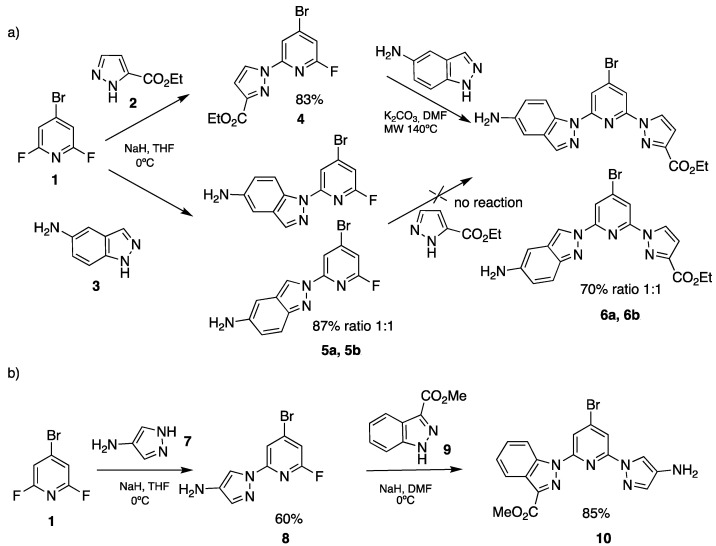
Synthesis of **ippy** derivatives.

When accessing these ligands with indazole and pyrazole both having different substituents, we have observed that the pyrazole ring must be introduced first, as the opposite monoindazole-substituted pyridine reduces or inactivates the reactivity of the remaining fluorine atom towards the entry of pyrazole. We have studied the synthesis of these **ippy** derivatives with 5-aminoindazole, 3-methoxycarbonylindazole, 4-amino, and 3-ethoxycarbonylpyrazole, as shown in Scheme 2.

In both cases, Scheme 2a,b, despite the electronic demand of the substituent on the pyrazole ring (amino or ethoxycarbonyl), this heterocycle must be introduced first to obtain **4** or **8**, as any of the monosubstituted indazolepyridine prepared inactivates or reduces the reactivity of the remaining fluorine atom. In the first synthesis (Scheme 2a), the introduction of 4-ethoxycarbonylpyrazole **2** to give **4** was followed by the introduction of 5-aminoindazole **3**, giving a mixture of regioisomers (**6a** and **6b**) in a 1:1 ratio. When the first substitution with 5-amino indazole **3** was carried out in the same way as pyrazole, a 1:1 mixture of both regioisomers (**5a** and **5b**) was also obtained, but attempts to carry out the second substitution with 3-ethoxycarbonylpyrazole did not work after several attempts with different solvents and temperatures. In the case of synthesis *b*, the same order was maintained. The first ring introduced was 4-amino pyrazole **7**, giving compound **8**, followed by the introduction of 3-methoxycarbonylindazole **9**, giving **ippy 10**. When 3-methoxycarbonylindazole was first introduced, the substitution of the remaining fluorine atom with a pyrazole ring was not possible.

**Scheme 3:**
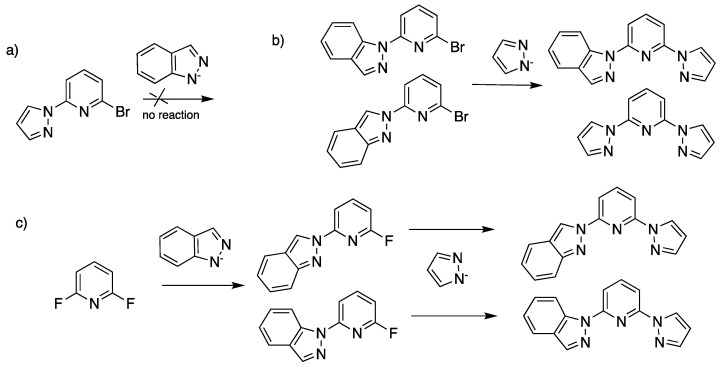
Halcrow’s results with unsubstituted pyrazole and indazole.

These results are in contrast to those reported by Halcrow and colleagues, where for the pristine pyrazole and indazole rings, the reaction to obtain **ippy** derivatives was only achieved from 2,6-dibromopyridine by the first substitution of bromine atoms with indazole and not with pyrazole (Scheme 3a,b) [22]. With 2,6-difluoropyridine (Scheme 3c) [23] as the starting material, a nonsymetrical ligand was also obtained if the first introduced heterocycle was indazole. It seems in these cases pyrazole hampered the introduction of the indazole in contrast to our results with substituted azoles.

Electronic demand of the substituents on the azole groups could be the cause of this apparent contradiction, although the behavior described is consistent with the opposite electronic demand caused by amino and methoxycarbonyl substituents. Until the more recent communication by Halcrow describing alternative synthetic access with 2,6-difluoropyridine, the lower reactivity of the bromine atoms or the good leaving effect of the indazolate (Scheme 3b) were the tentative explanations for this behavior. 

### 2.2. Bipyrazole Pyridine (***bppy***)

**Scheme 4:**
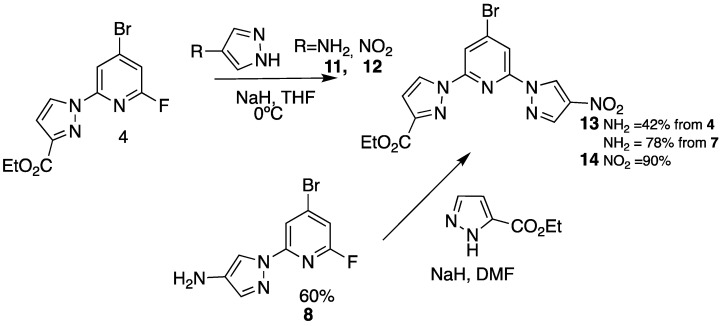
Synthetic access to **bppy** derivatives.

It is possible to obtain **bppy** derivatives from a first substitution with 3-ethoxycarbonylpyrazole, followed by a second substitution with any of the attempted 4-amino **11** and 4-nitro **12** pyrazoles. However, for the 4-aminopyrazole, the yield was only moderate (42%), whereas with 4-nitropyrazole in more gentle conditions, the **bppy** derivative was obtained in 90% yield. Due to the different electronic demands of both groups on the pyrazolate salt and the starting fluoro monosubstituted pyridine, access to this amino **bppy** was better performed in yields by the first introduction of 4-aminopyrazole, followed by a second substitution with 3-ethoxycarbonylpyrazole (Scheme 4).

### 2.3. Biindazole Pyridine (***bipy***)

**Scheme 5:**
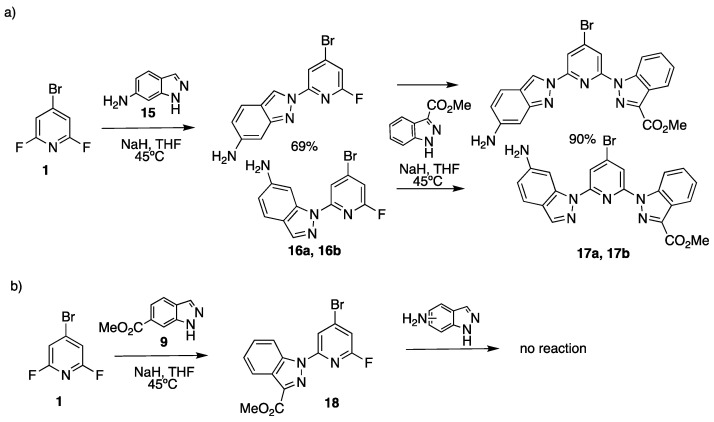
Synthetic access to **bipy** derivatives.

The nonsymmetrical **bipy** compounds with 5-aminoindazole, 6-aminoindazole, and 3-methoxycarbonylindazole were obtained in the first step by the introduction of the 5- or 6-aminoindazole (represented in Scheme 5a), followed by the second step of substitution of the remaining fluorine atoms with 3-methoxycarbonylindazole. The substitution with both amino compounds (those obtained from 6-aminoindazole are the only represented in Scheme 5a, as both isomers **16a-b** or **17a-b** could be separated) drove both possible regioisomers in a 1:1 ratio. The opposite order gave no reaction when 3-methoxycarbonylindazol **9** was firstly introduced, compound **18**. The reaction of **18** with 5- or 6-aminoindazole was attempted in different solvents and at different temperatures, including microwave-mediated synthesis (Scheme 5b). 

**Scheme 6:**
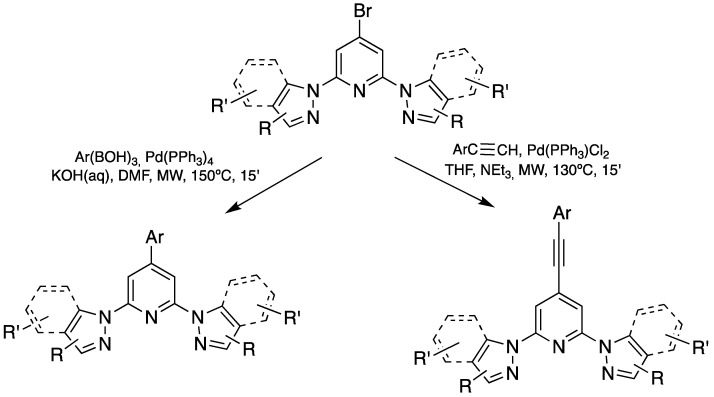
Synthesis of 4-substituted pyridine by Suzuki and Sonogashira reactions.

Out of all nonsymmetrical (**ippy**, **bppy**, and **bipy**) compounds described, the bromine atom on the 4-position of pyridine was functionalized by a Suzuki and Sonogashira palladium(0) cross-coupling reaction with different arylboronic acids and arylacetylene derivatives following the general procedures described in Scheme 6.

**Scheme 7:**
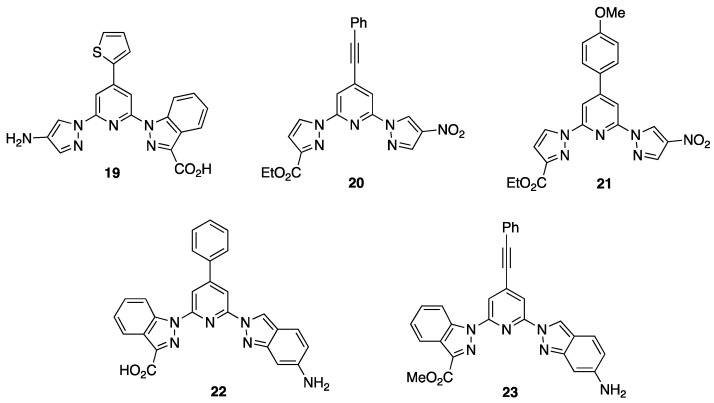
Examples of molecules prepared from Pd-coupling reactions.

Suzuki- and Sonogashira-type reactions were carried out under microwave irradiation in only 15′, with yields comparable to those under normal reflux conditions. In Suzuki reactions, it is also possible to use KOH (1 M) to obtain the hydrolyzed carboxylic acids, which is very convenient when the resultant amino acids are important to their complexing capabilities (for instance, towards lanthanide ions, as they are one of our main interests). Four examples for these coupling reactions are described for obtaining the compounds depicted in Scheme 7.

The possibility of making the bromine Pd(0) cross-coupling reaction in any of the other two synthetic steps, over the starting trihalopyridine or over the monopyrazole or monoindazole derivatives, was proved affordable without detrimental yielding results in some of the intermediate compounds described.

## 3. Conclusions and Outlook

In summary, we have shown that by means of control of the reaction conditions it is possible to access 2,4,6-nonsymmetricaly trisubstituted pyridines from readily accessible 4-bromo-2,6-difluoropyridine. The opportunities of this tri-functionalization are almost endless, and it allows for the ability to tune the electronic π character of these trisheterocyclic units in order to obtain the best candidates for ligands in a variety of transition and lanthanide ions complexes.

## 4. Materials and Methods

### 4.1. General Methods

All solvents (THF and DMF) were purified before use and kept dry over molecular sieves. All other reagents were of reagent grade and used as received. ^1^H-NMR, ^13^C-NMR: Bruker AV-300 spectrometer (Departamento de Química Orgánica, QO). MS: VG Autospec in EI, FAB and FAB-HRMS modes (L-SIMS^+^), ABSciex QSTAR (ESI^+^, HRMS), Bruker ULTRAFLEX III (MALDI-TOF/TOF) (HRMS), spectrometers (Servicio Interdepartamental de Investigación, SIdI). Elemental analyses: Perkin-Elmer CHN 2400 automatic analyzers (SIdI). 

### 4.2. General Synthetic Procedures

#### 4.2.1. Substitution of Fluorine Atoms by Pyrazolates or Indazolates: Method A: NaH in THF

In a round-bottom Schlenk flask under argon, the corresponding fluoropyridine (5.15 mmol) and azole (5.15 mmol) compounds were dissolved in 25 mL of dry THF. The solution was cooled to 0 °C, and NaH (60% in oil, 226 mg, 5.15 mmol) was added. The reaction mixture was left at rt for 4 h, after which 15 mL of CH_2_Cl_2_ was added. The organic phase was washed with water (15 mL), and the aqueous phase was extracted with CH_2_Cl_2_ (3 × 10 mL). The combined organic phases were dried over Na_2_SO_4_ and concentrated to dryness, giving a solid that was purified by flash chromatography (CH_2_Cl_2_/hexane 1:1) to a white solid.

#### 4.2.2. Substitution of Fluorine Atoms by Pyrazolates or Indazolates: Method B: NaH in DMF 

In a round-bottom Schlenk flask under argon, the corresponding fluoropyridine (3.5 mmol) and azole (8.3 mmol) were dissolved in 30 mL of freshly distilled DMF. The solution was cooled to 0 °C, and NaH (60% in oil, 364 mg, 9.1 mmol) was added. The reaction mixture was left at rt, then heated at 40 °C for 5 h, after which a white precipitate formed. The solid was isolated by centrifugation, and the addition of water to the liquid phase gave a second precipitate, which was also isolated by centrifugation. The combined solids were washed with warm hexane (40 mL) and water (40 mL).

#### 4.2.3. Substitution of Fluorine Atoms by Pyrazolates or Indazolates: Method C: K_2_CO_3_ in DMF and Microwave Radiation

To a microwave vial under argon contained substituted monofluorinated 4-bromopyridine (1 eq.), 3-methoxycarbonyl indazole (1.2 eq.), K_2_CO_3_ (1.2 eq.), and dry recently distilled DMF (3 mL). The mixture was stirred at room temperature for 2 min and then heated at 140 °C for 2 h in a microwave oven (Biotage Initiator 2.5). The reaction mixture was poured on 15 mL of water and the resulting solid was isolated by centrifugation and washed 3 times with 10 mL of water. The product was purified by Flash chromatography in CH_2_Cl_2_:MeOH in a 95:5.

#### 4.2.4. Microwave-Assisted Suzuki-Miyaura Cross-Coupling Reaction with Boronic Acids

To a microwave vial under argon containing substituted 4-bromopyridine (0.5 mmol), the corresponding boronic acid (0.51 mmol) and Pd(PPh_3_)_4_ (0.02 mmol) were dissolved in anhydrous THF (3.5 mL), and were stirred at room temperature for 10 min. Then, a solution of 0.5 mL KOH (1 M) was added to the mixture. The mixture was heated at 130 °C for 11 min in a microwave oven (Biotage Initiator 2.5). The solvent was removed under vacuum to yield a black residue, which was dissolved in 20 mL of CH_2_Cl_2_ and washed with 10 mL of water. The aqueous phase was extracted with CH_2_Cl_2_ (2 × 20 mL). The combined organic phases were washed with brine (20 mL), dried over Na_2_SO_4_, filtered, and concentrated to dryness. Flash chromatography in CH_2_Cl_2_:hexane in a 1:1 ratio yielded the corresponding trisubstituted 2,4,6-pyridine compounds.

#### 4.2.5. Microwave-Assisted Sonogashira Cross-Coupling Reaction with Phenylacetylene

In a microwave vial under argon containing substituted 4-bromopyridine (0.46 mmol), PdCl_2_ (PPh_3_)_2_ (0.061 eq.) and CuI (0.061 eq.) were dissolved in a mixture of anhydrous THF (2.5 mL) and freshly distilled NEt_3_ (2.2 mL), and were stirred for 5 min. Then, phenylacetylene (1.2 eq.) was added, and the mixture was heated at 120 °C for 13 min in a microwave oven (Biotage Initiator 2.5). The solvent was removed under reduced pressure to yield a dark residue, which was dissolved in 20 mL of CH_2_Cl_2_ and washed with 15 mL of water. The aqueous phase was extracted with CH_2_Cl_2_ (2 × 20 mL). The combined organic phases were washed with brine (20 mL), dried over Na_2_SO_4_, vacuum filtered, and concentrated to dryness. Flash column chromatography, CH_2_Cl_2_:hexane 9:1, yielded the corresponding trisubstituted 2,4,6-pyridine compounds. 

### 4.3. Chemical Synthesis and Characterization

*2-(3-Ethoxycarbonyl-1-pyrazolyl)-4-bromo-6-fluoropyridine* (**4**). Obtained by general method A from 4-bromo-2,6-difluoropyridine **1** (2.77 g, 14.28 mmol) and 3-ethoxycarbonylpyrazol **2** (1 g, 7.14 mmol). **4** was obtained as a white solid (1.86 g, 83%). ^1^H-NMR (CDCl_3_) δ (ppm) (300 MHz): 8.46 (d, *J* = 2.7 Hz, 1H); 8.21 (dd, *J* = 1.3, 1.10 Hz, 1H); 7.06 (dd, *J* = 2.7 Hz, 2.59 Hz, 1H); 6.96 (d, *J* = 2.7 Hz, 1H); 4.44 (c, *J* = 7.1 Hz, 2H); 1.42 (t, *J* = 7.1 Hz, 3H). ^13^C-NMR (CDCl_3_) δ (ppm) (75 MHz): 163.7, 161.7, 160.5, 147.0, 137.9, 129.0, 113.4, 111.0, 110.7, 61.8, 14,3. Anal. Calc. for C_11_H_9_BrFN_3_O_2_: C 42.06, H 2.89, N 13.38, found: C 42.43, H 3.16, N 13.18. MS (FB^+^): *m*/*z* = 314.0 ([M]^+^, 100%), 316.0 ([M]^+^, 98%).

*2-(5-Aminoindazolyl)-4-bromo-6-fluoropyridine* (**5a**, **5b**). Obtained by general method C from 4-bromo-2,6-difluorpyridine **1** (150 mg, 0.478 mmol) and 5-aminoindazole **3** (71 mg, 0.573 mmol). A non-separable mixture of **5ab** was obtained as a white solid (13 mg, 64%). The ^1^H-RMN spectra were assigned from a small amount of **5a** isolated after preparative HPLC. ^1^H-NMR (CDCl_3_) δ (ppm) (300 MHz): Isomer **5a**: 8.53 (d, *J* = 8.8 Hz, 1H); 8.06 (s, 1H); 8.00 (s, 1H); 6.97 (d, *J* = 2.2 Hz, 1H); 6.95–6.93 (m, 1H); 6.86 (m, 1H). Isomer **5b**: 8.64 (s, 1H); 8.27 (s, 1H); 7.54 (d, *J* = 9.20 Hz, 1H); 7.03 (m, 1H); 6.89 (d, *J* = 2.2 Hz, 1H); 6.71 (m, 1H). Anal. Calc. for C_12_H_8_BrFN_4_: C 46.93, H 2.63, N 18.24, found: C 46.73, H 2.46, N 18.18. MS (FAB^+^): *m*/*z* = 306.0 ([M]^+^, 100%); 308.0 ([M]^+^, 97%). 

*2-(5-Aminoindazolyl)-4-bromo-6-(3-ethoxycarbonyl-1-1pyrazolyl)pyridine* (**6a**, **6b**). Obtained by general method A from 4-bromo-2-(3-ethoxycarbonyl-1-pyrazolyl)-6-fluoropyridine **4** (2.77 g, 14.28 mmol) and 5-aminoindazol **3** (1 g, 7.14 mmol). **6a, 6b** was obtained as a white solid (1.86 g, 83%). ^1^H-NMR (CDCl_3_) δ (ppm) (300 MHz): Isomer **6a**: 8.55 (d, *J* = 2.5 Hz, 1H); 8.38 (d, *J* = 8.7 Hz, 1H); 8.13 (d, *J* = 1.2 Hz, 1H); 8.08 (d, *J* = 1.2 Hz, 1H); 8.01 (s, 1H); 7.04–6.95 (m, 4H); 6.87 (dd, *J* = 1.9 Hz, 8.7 Hz, 1H); 4.47 (c, *J* = 7.1 Hz, 4H); 1.45 (t, *J* = 7.1 Hz, 6H). Isomer **6b**: 8.68 (m, 1H); 8.60 (d, *J* = 2.6 Hz, 1H); 8.31 (d, *J* = 1.2 Hz, 1H); 8.23 (d, *J* = 1.2 Hz, 1H); 7.55 (d, *J* = 9.2 Hz, 1H); 7.04–6.95 (m, 4H); 6.71 (d, *J* = 1.2 Hz, 1H); 4.47 (c, *J* = 7.1 Hz, 4H); 1.45 (t, *J* = 7.1 Hz, 6H). Anal. Calc. for C_18_H_15_BrN_6_O_2_: C 50.60, H 3.54, N 19.67, found: C 50.73, H 3.46, N 19.56. MS (FAB^+^): *m*/*z* = 427.0 ([M]^+^, 100%); 429.0 ([M]^+^, 98%).

*2-(4-Amino-1H-pyrazolyl)-4-bromo-6-fluoropyridine* (**8**). Obtained by general method A from 4-bromo-2,6-difluoropyridine **1** (1.05 mmol) and 4-aminopyrazole **7** (1 mmol). **8** was obtained as a white solid (60%). ^1^H-RMN (CDCl_3_) δ (ppm) (300 MHz): 7.98 (s, 1H); 7.97 (s, 1H); 7.47 (s, 1H); 6.93 (s, 1H). ^13^C-NMR (CDCl_3_) δ (ppm) (75 MHz): 137.13–137.01, 136.30, 131.90, 113.75, 111.67, 111.61, 108.5, 107.99. Anal. Calc. for C_8_H_6_BrFN_4_: C 37.38, H 2.35, N 21.80, found: C 38.34, H 2.44, N 21.80. MS (FAB^+^): *m*/*z* = 256.0 ([M]^+^, 100%), 258.0 ([M]^+^, 97%).

*2-(3-Methoxycarbonyl-1H-indazolyl)-4-bromo-6-(4-amino-1H-pyrazolyl)pyridine* (**10**). Obtained by general method A from 2-(4-amino-1H-pyrazolyl)-4-bromo-6-fluoropyridine **8** (1 mmol) and 3-methoxycarbonylindazole **9** (1.1 mmol). **10** was obtained as a white solid (84%). ^1^H-NMR (CDCl_3_) δ (ppm) (300 MHz): 8.67 (d, 1H, *J* = 7.29 Hz); 8.31 (d, 1H, *J* = 8.22 Hz); 8.14 (s, 1H); 8.05 (s, 1H); 8.01 (s, 1H); 7.62 (t, 1H, *J* = 7.29 Hz); 7.48 (s, 1H); 7.45 (t, 1H, *J* = 7.34 Hz); 4.10 (s, 3H). ^13^C-NMR (CDCl_3_) δ (ppm) (75 MHz): 162.54, 152.18, 150.59, 139.99, 138.54, 136.03, 134.32, 131.88, 128.78, 125.04, 124.73, 119.44, 116.90, 113.73, 112.06, 108.28, 52.48. Anal. Calc. for C_17_H_13_BrN_6_O_2_: C 49.41, H 3.17, N 20.34, found: C 49.34, H 3.24, N 20.30. MS (FAB^+^): *m*/*z* = 412.0 ([M]^+^, 100%), 414.0 ([M]^+^, 97%).

*2-(3-Ethoxycarbonyl-1H-pyrazolyl)-4-bromo-6-(4-amino-1H-pyrazolyl)pyridine* (**13**). Obtained by general method A from 2-(3-ethoxycarbonyl-1H-pyrazolyl)-4-bromo-6-fluoropyridine **4** (0.46 mmol) and 4-aminopyrazole **7** (0.5 mmol). **13** was obtained as a white solid (42%). Alternatively can also be prepared from 2-(4-amino-1H-pyrazolyl)-4-bromo-6-fluoropyridine **8** and 3-ethoxycarbonylpyrazole **2** following general method A (yield 78%). ^1^H-NMR (CDCl_3_ 300 MHz) δ: 8.51 (1H, d, *J* = 2.27 Hz); 8.09 (1H, s); 8.00 (1H, s); 7.43 (1H, s); 6.98 (1H, s); 4.45 (2H, c, *J* = 7.18 Hz); 1.43 (3H, t, *J* = 7.18 Hz). ^13^C-NMR (CDCl_3_ 75 MHz) δ: 161.86, 150.58, 149.6, 146.57, 141.66, 136.55, 136.15, 128.47, 113.46, 113.03, 112.26, 110.41, 61.44, 14.32 ppm. Anal. Calc. for C_14_H_13_BrN_6_O_2_: C 44.58, H 3.47, N 22.28, found: C 44.39, H 3.52, N 22.32. MS (FAB^+^): *m*/*z* = 376.0 ([M]^+^, 100%), 378.0 ([M]^+^, 97%).

*2-(3-Ethoxycarbonyl-1H-pyrazolyl)-4-bromo-6-(4-nitro-1H-pyrazolyl)pyridine* (**14**). Obtained by general method A from 2-(3-ethoxycarbonyl-1H-pyrazolyl)-4-bromo-6-fluoropyridine **4** (1 mmol) and 4-nitropyrazole **12** (1 mmol). **14** was obtained as a white solid (90%). ^1^H-NMR (CDCl_3_ 300 MHz) **δ**: 9.19 (1H, s); 8.56 (1H, d, *J* = 2.63 Hz); 8,38 (1H, d, *J =* 1.32 Hz); 8.30 (1H, s); 8.16 (1H, d, *J* = 1.32 Hz); 7.04 (1H, d, *J* = 2.63 Hz); 4.47 (2H, c, *J* = 7.24 Hz); 1.44 (3H, t, *J* = 7.23 Hz) ppm. ^13^C-NMR (CDCl_3_ 75 MHz) **δ**: 171,47, 161.39, 149.15, 146.11, 136.09, 135.69, 131.37, 128.01, 113.03, 112.57, 111.81, 109.96, 60.98, 13.87 ppm. Anal. Calc. for C_14_H_11_BrN_6_O_4_: C 41.30, H 2.72, N 20.64, found: C 41.39, H 2.42, N 20.63. FAB-HRMS *m*/*z* found: 406.0034, calculated for C_14_H_11_BrN_6_O_4_: 406.0025.

*2-(6-Aminoindazolyl)-4-bromo-6-fluoropyridine* (**16a**, **16b**). Obtained by general method A from 4-bromo-2,6-difluorpyridine **1** (1 g, 5.26 mmol) and 6-aminoindazole **15** (700,4 mg, 5.26 mmol). The resulting solid was purified by Flash chromatography (dichloromethane: MeOH, 99:1). **16a** was a white solid (450 mg, 28%) and **16b** a yellow solid (430 mg 27%).

Isomer **16a.** ^1^H-NMR (CDCl_3_) δ (ppm) (300 MHz): 8.07 (s, 1H); 8.00 (s, 1H); 7.98 (d, *J* = 1.1 Hz, 1H); 7.49 (d, *J* = 8.5 Hz, 1H); 6.87 (dd, *J* = 1,1 Hz, 2.6 Hz, 1H); 6.68 (dd, *J* = 1.9 Hz, 8.5 Hz, 1H). ^13^C-NMR (CDCl_3_) δ (ppm) (300 MHz): 162.1, 154.5, 147.8, 140.6, 138.4, 136.6, 121.8, 119.5, 113.8, 113, 106.8, 98.7. Anal. Calc. for C_12_H_8_BrN_4_: C 46.93, H 2.63, N 18.24, found: C 46.79, H 2.81, N 18.03. MS (FAB^+^): *m*/*z* = 307.0 ([M]^+^, 100%); 309.0 ([M]^+^, 97%).

Isomer **16b.** ^1^H-NMR (CDCl_3_) δ (ppm) (300 MHz): 8.78 (s, 1H); 8.25 (s, 1H); 7.49 (d, *J* = 8.9 Hz, 1H); 7.01 (d, *J* = 1.4 Hz, 1H); 6.70 (s, 1H); 6.60 (dd, *J* = 1.4 Hz, 8.9 Hz, 1H). ^13^C-NMR (CDCl_3_) δ (ppm) (300 MHz): 162.1, 150.4, 146.6, 152.5, 137.6, 121.4, 122.3, 118.4, 118, 113.5, 109.9, 95.5. Anal. Calc. for C_12_H_8_BrN_4_: C 46.93, H 2.63, N 18.24, found: C 47.02, H 2.81, N 18.11. MS (FAB^+^): *m*/*z* = 307.0 ([M]^+^, 100%); 309.0 ([M]^+^, 97%).

*2-(6-Aminoindazolyl)-4-bromo-6-(3-methoxycarbonyl-1H-indazolyl)pyridine* (**17a**). Obtained by general method A from **16a** (1.16 mmol) and 3-methoxycarbonylindazole **9** (1.166 mmol). **9a** was obtained as a white solid (478 mg. 89%). ^1^H-NMR (CDCl_3_) δ (ppm) (300 MHz): 8.69 (d, *J* = 8.5 Hz, 1H); 8.29 (d, *J* = 8.1 Hz, 1H); 8.11 (d, *J* = 1.3 Hz, 1H); 8.05 (s, 1H); 7.99 (d, *J* = 1.3 Hz, 1H); 7.97 (s, 1H); 7.58 (m, 1H); 7.55–7.53 (m, 1H); 7.46–7.41 (m, 1H); 6.70 (dd, *J* = 1.8 Hz, 8.5 Hz, 1H); 4.10 (s, 3H). ^13^C-NMR (CDCl_3_) δ (ppm) (300 MHz): 163.7, 152.3, 151.2, 148.2, 144.4, 142.6, 137.2, 135.2, 128.9, 126.6, 123.1, 122.9, 122.7, 119.4, 118.6, 113.6, 112.7, 110.9, 108.7, 88.4, 53.0. Anal. Calc. for C_21_H_15_BrN_6_O_2_: C 54.44, H 3.26, N 18.14, found: C 54.10, H 3.33, N 18.31. MS (FAB^+^): *m*/*z* = 463.0 ([M]^+^, 100%); 465.0 ([M]^+^, 97%).

*2-(3-Carboxymethyl-1H-indazolyl)-4-bromo-6-fluoropyridine* (**18**). Obtained by general method A from 4-bromo-2,6-difluorpyridine **1** (1 g, 5.15 mmol) and 3-carboxymethylindazole **9** (900 mg, 5.11 mmol). **18** was purified by chromatography (Hexane:dichloromethane 3:1) and isolated as a white solid (1.5 g, 84%). ^1^H-NMR (CDCl_3_) δ (ppm) (300 MHz): 8.78 (d, *J* = 8.5 Hz, 1H); 8.28–8.26 (m, 2H); 7.60 (t, *J* = 7.5 Hz, 1H); 7.44 (t, *J* = 7.5 Hz, 1H); 7.04 (d, *J* = 3.0 Hz, 1H); 4.10 (s, 3H). ^13^C-NMR (CDCl_3_) δ (ppm) (300 MHz): 163.7, 161.8, 159.7, 157.2, 142.0, 137.2, 131.4, 126.6, 122.9, 121.7, 119.4, 114.0, 108.5, 107.4, 53.0. Anal. Calc. for C_21_H_15_BrN_6_O_2_: C 54.44, H 3.26, N 18.14, found: C 54.10, H 3.33, N 18.31. MS (ESI^+^): *m*/*z* = 350.0 ([M]^+^, 100%), 352.0 ([M]^+^, 97%).

*2-(3-Carboxy-1H-indazolyl)-4-(2-tienyl)-6-(4-amino-1H-pirazolil) piridina* (**19**). Obtained by microwave-assisted Suzuki–Miyaura cross-coupling reaction with boronic acids from **10** (100.2 mg, 0.243 mmol) and tienylboronic acid (34 mg, 0.227 mmol). The product in the form of carboxylic acid **11** was isolated by centrifugation and washed with dichloromethane (60%). ^1^H-NMR (DMSO, 300 MHz) **δ**: 8.72 (1H, d, *J* = 8.29 Hz); 8.39 (1H, d*, J* = 7.91 Hz); 8.32 (1H, d*, J* = 1.51 Hz); 8.19 (1H, s); 7.90 (1H, d, *J* = 1.13 Hz); 7.85 (1H, d*, J* = 3.77 Hz); 7.61 (1H, d, *J* = 4.14 Hz); 7.58 (1H, t, *J* = 7.16 Hz); 7.52 (1H, s); 7.36 (1H, t*, J* = 7.54 Hz); 7.21 (1H, dd, *J*_1_ = 4.90, *J*_2_ = 3.77 Hz) ppm. ^13^C-NMR (DMSO,75 MHz) **δ:** 153.5, 152.2, 151.6, 149.3, 139.7, 139.1, 137.6, 136.4, 130.3, 129.1, 128.9, 128.2, 127.6, 127.1, 122.9, 122.1, 117.32, 114.8, 109.2, 106.4 ppm. Anal. Calc. for C_20_H_14_N_6_O_2_S: C 59.69, H 3.51, N 20.88, found: C 59.40, H 3.61, N 20.53. MS (ESI^+^): *m*/*z* = 403.9 ([M+H]^+^, 100%).

*2-(3-Ethoxycarbonyl-1H-pyrazolyl)-4-phenylethynyl-6-(4-nitro-1H-pyrazolyl)pyridine* (**20**). Obtained by microwave-assisted Sonogashira cross-coupling reaction with phenylacetylene from **13** (150 mg, 0.36 mmol) and phenylacetylene (0.39 mmol). The product was purified by Flash chromatography (gradient dichlorometane/hexane (20–100%)) yield 80%. ^1^H-NMR (CDCl_3_, 300 MHz) δ: 9.19 (1H, s); 8.57(1H, d, *J* = 2.63 Hz); 8.27 (1H, s); 8.23 (1H, s); 8.00 (1H, s); 7.58–7.54 (2H, m); 7.43–7.37 (3H, m); 7.01 (1H, d, *J* = 2.64 Hz); 4.45 (2H, c, *J* = 7.02 Hz); 1.44 (3H, t, *J* = 7.01 Hz) ppm. ^13^C-NMR (CDCl_3_, 75 MHz) δ: 162.0, 151.3, 149.4, 136.5, 135.1, 134.9, 133.5, 132.1 (2C), 132.1, 129.5, 128.4 (2C), 128.2, 121.8, 113.7, 111.8, 110.9, 110.2, 95.3, 86.4, 61.0, 14.4 ppm. Anal. Calc. for C_22_H_16_N_6_O_4_: C 61.68, H 3.76, N 19.62, found: C 61.82, H 3.39, N 19.69. MS (ESI^+^): *m*/*z* = 429.1 ([M + H]^+^, 100%).

*2-(3-Ethoxycarbonyl-1H-pyrazolyl)-4-(phenyl-p-methoxy)-6-(4-nitro-1H-pyrazolyl)pyridine* (**21**). Obtained by microwave-assisted Suzuki–Miyaura cross-coupling reaction with boronic acids from **13** (150 mg, 0.36 mmol) and *p*-metoxyphenylboronic acid (0.4 mmol). The product was isolated as a white solid 78 mg, 48% yield). ^1^H-NMR (CDCl_3_ 300 MHz) δ: 9.26 (1H, s); 8.63 (1H, d, *J* = 2.69 Hz); 8.35 (1H, d, *J* = 1,25 Hz); 8.31 (1H, s); 8.18 (1H, d, *J* = 1.32 Hz); 7.80 (2H, d, *J* = 8.86 Hz); 7.05 (2H, d, *J* = 8.86 Hz); 7.04 (1H, d, *J* = 2.69 Hz); 4,47 (2H, c, *J* = 7.10 Hz); 3.89 (3H, s); 1.45 (3H, t, *J* = 7.10 Hz) ppm. ^13^C-NMR (CDCl_3_, 75 MHz) δ: 161.44, 161.16, 155.32, 154.36, 149.84, 148.55, 136.98, 131.30, 128.28, 128.20, 128.08, 125.45, 114.24, 110.15, 108.92, 107.78, 61.00, 55.02, 13.87 ppm. Anal. Calc. for C_21_H_18_N_6_O_5_: C 58.06, H 4.18, N 19.35, found: C 57.23, H 3.89, N 19.29. MS (ESI^+^): *m*/*z* = 435.1 ([M + H]^+^, 100%).

*2-(6-Amino-1H-indazoyil)-4-phenyl-6-(3-carboxy-1H-indazolyl)pyridine* (**22**). Obtained by microwave-assisted Suzuki–Miyaura cross-coupling reaction with boronic acids from **17a** (0.15 mmol) and phenylboronic acid (0.17 mmol). The product in the form of carboxylic acid **22** was isolated by centrifugation and washed with dichloromethane (89% yield). ^1^H-NMR (CD_3_OD) δ (ppm) (300 MHz): 9.11 (s, 1H); 8.29 (d, *J* = 8.5 Hz, 1H); 8.05 (m, 2H); 7.93 (s, 1H); 7.73 (d, *J* = 8.5 Hz, 1H); 7.55–7.52 (m, 2H); 7.41 (m, 1H); 7.36 (m, 1H); 7.28–7.24 (m, 3H); 7.16 (m, 2H). ^13^C-NMR (CD_3_OD) δ (ppm) (300 MHz): 165.7, 153.8, 153.5, 152.4, 140.7, 139.4, 139.0, 138.9, 138.8, 136.4, 131.7, 131.0, 130.0, 127.4, 127.2, 125.9, 125.1, 124.3, 123.8, 118.6, 115.0, 111.7, 110.6, 108.8. Anal. Calc. for C_26_H_18_N_6_O_2_: C 69.95, H 4.06, N 18.82, found: C 70.02, H 3.98, N 19.11. MS (FAB^+^): *m*/*z* = 446.1 ([M]^+^, 100%).

*2-(6-Amino-1H-indazolyl)-4-phenylethinyl-6-(3-methoxycarbonyl-1H-indazolyl)pyridine* (**23**). Obtained by microwave-assisted Sonogashira cross-coupling reaction with phenylacetylene from **17a** (0.15 mmol) and phenylacetylene (0.17 mmol). The product was purified by Flash chromatography (gradient dichlorometane/hexane (20–100%)) yield (49 mg, 67%). ^1^H-NMR (CDCl_3_) δ (ppm) (300 MHz): 8.74 (d, *J* = 8.5 Hz, 1H); 8.31 (d, *J* = 8.1 Hz, 1H); 8.05–8.03 (m, 3H); 7.94 (s, 1H); 7.59–7.51 (m, 4H); 7.43–7.39 (m, 4H); 6.72 (dd, *J* = 1.6 Hz, 8.5 Hz, 1H); 4.11 (s, 3H). ^13^C-NMR (CDCl_3_) δ (ppm) (300 MHz): 163.7, 151.6, 150.5, 148.2, 137.2, 135.2, 133.8, 132.0, 131.7, 128.8, 128.6, 128.1 126.6, 124.0, 124.0, 122.9, 122.7, 119.6, 119.4, 116.2, 114.4, 113.6, 109.7, 91.9, 91.2, 89.3, 53.0. Anal. Calc. for C_29_H_20_N_6_O_2_: C 71.89, H 4.16, N 17.35, found: C 71.76, H 3.98, N 17.21. MS (FAB^+^): *m*/*z* = 484.1 ([M]^+^, 100%).

## Data Availability

Analytical data can be found in the archives of The Sericio Interdepartamental de Investigación of the University Autónoma de Madrid, https://www.uam.es/uam/sidi (accessed on 15 February 2022).

## Sample Availability

Samples of the final compounds **19**–**23** are available from the authors.
